# The association between income inequality and all-cause mortality across urban communities in Korea

**DOI:** 10.1186/s12889-015-1924-x

**Published:** 2015-06-20

**Authors:** Jong Park, So-Yeon Ryu, Mi-ah Han, Seong-Woo Choi

**Affiliations:** Department of Preventive Medicine, Chosun University Medical School, 309, Pilmun-daero, Gwangju, 501-759 Republic of Korea

## Abstract

**Background:**

Korea has achieved considerable economic growth more rapidly than most other countries, but disparities in income level have increased. Therefore, we sought to assess the association between income inequality and mortality across Korean cities.

**Methods:**

Data on household income were obtained from the 2010-2012 Korean Community Health Survey and data on all-cause mortality and other covariates were obtained from the Korean Statistical Information Service. The Gini coefficient, Robin Hood index, and income share ratio between the 80th and 20th percentiles of the distribution were measured for each community. After excluding communities affected by changes in administrative districts between 2010 and 2012, a total of 157 communities and 172,398 urban residents were included in the analysis.

**Results:**

When we graphed income inequality measures versus all-cause mortality as scatter plots, the R square values of the regression lines for GC, RHI, and 80/20 ratios relative to mortality were 0.230, 0.238, and 0.152, respectively. After adjusting for other covariates and median household income, mean all-cause mortality increased significantly with increasing GC (P for trend = 0.014) and RHI (P for trend = 0.031), and increased marginally with 80/20 ratio (P for trend = 0.067).

**Conclusions:**

Our data demonstrate that income inequality measures are significantly associated with all-cause mortality rate after adjustment for covariates, including median household income across urban communities in Korea.

## Background

In the past four decades, there has been great interest in the relationship between income inequality and various health indicators including life expectancy [1], self-rated health status [2] and mortality [3]. Research so far suggests that not only does absolute income influence health but that the inequality of income distribution also has an impact [4]. While this association has been studied across a wide range of countries [5, 6], whether income data are comparable and complete across all samples remains in question [3]. Others have investigated the effect of income inequality on health across regions within countries. However, most of these studies have been conducted in developed countries such as the United States [7], Canada [8], the United Kingdom [9], other European countries [10, 11] and Japan [12]. Furthermore, their results are not in agreement.

Korea has achieved considerable economic growth more rapidly than most other countries, but disparities in income and education have increased, especially after the economic crisis in the late 1990s [13]. Furthermore, health status varies greatly across regions because of rapid urbanization. According to the Korean Statistical Information Service (KSIS), the region with the highest all-cause mortality in 2012 was Jeollanam-do (436.0 per 100,000) and the lowest was Seoul (339.7 per 100,000) [14]. However, to our knowledge, few studies have investigated how the association of mortality with income inequality varies by region in Korea. As mentioned above, since most studies have been conducted using post-industrialized countries’ data, investigating the relationship between income inequality and health in emerging nations like Korea may yield new insights. Thus, we sought to assess the association between income inequality and mortality across urban communities in Korea.

## Methods

### Sources of data

Data on household income were from Korean Community Health Survey (KCHS) and data on mortality rate and control variables (community population, percent of over 65 years in community population, ratio of social welfare expenditure to the general budget, number of physicians per 1,000 individuals, smoking rate, and drinking rate) in 2010–2012 were from KSIS.

Data on household income were obtained from the 2010–2012 KCHS conducted in 253 communities: 167 urban communities and 86 rural communities. KCHS is a nationwide health interview survey carried out by the Korean Centers for Disease Control and Prevention (KCDC) to estimate patterns of disease prevalence and morbidity, as well as to understand the lifestyle and health behaviors of adults aged 19 years and over [15]. The KCHS uses a multistage sampling design to obtain a representative sample. Within each of the 253 communities, 90 primary sampling units (PSUs) corresponding to smaller geographic entities were randomly selected; this was followed by the random selection of five to eight households within the PSU and in-person interviews with all adults in those households [15]. The KCHS database of surveys from 2010–2012 contained pooled data from 687,376 interviews (2010: 229,229; 2011: 229,226; 2012: 228,921). For this study to collect one household income data in each household, the first members of each household (338,045 total subjects) were considered, and among them, residents in cities (206,013) were selected. We excluded 16,908 individuals due to insufficient information on income and excluded 16,707 whose household income was less than the 5th percentile or higher than the 95th percentile in their community to remove outliers and extreme values*.* Finally, a total of 172,398 individuals were included in the analysis. The protocol of community health survey was reviewed and approved by the Institutional Review Board of KCDC (2010–02–CON–22–P). Written informed consent was obtained from all participants in the community health survey.

Data on mortality rate and other covariates in 2010–2012 were obtained from KSIS. KSIS collected various financial and social data on 250 communities: 167 urban communities and 83 rural communities. The data evaluated included: a) age-standardized all-cause mortality per 100,000 individuals, b) community population, c) percent of over 65 years in community population (%), d) ratio of social welfare expenditure to the general budget (%), e) number of physicians per 1,000 individuals, f) smoking rate, and g) drinking rate.

KCHS and KSIS definitions of urban communities differed; in at least one instance, a community considered a single entity by KCHS was defined as two by KSIS. Furthermore, boundaries and names of some administrative districts changed from 2010 to 2012. For example, Yeongi-gun became Sejong city, the administrative capital, and Changwon city was consolidated with Masan and Jinhae. After excluding communities whose definitions differed between databases or changed over the study period, a total of 157 urban communities were included.

### Measure of household income and income inequality

Household income was measured through KCHS and was defined as income before payment of taxes and receipt of benefits. We used three alternative measures of income distribution: the Gini coefficient (GC), the Robin Hood index (RHI), and the income share ratio between the 80th and 20th percentiles of the distribution (80/20 ratio). The GC is calculated as the ratio of the area between the actual income distribution (the Lorenz curve) and the diagonal enclosing the distribution curve to the total area under the diagonal. Higher GCs mean greater income inequality and range from 0, meaning perfect equality, to 1, perfect inequality [3]. The RHI was estimated from state-specific data on share of total household income arranged by tenths of the distribution. This value approximates the share of total income that has to be taken from those above the mean and transferred to those below the mean to achieve equality in the distribution of incomes. The higher the value of the index, the less egalitarian is the distribution of income [16]. The 80/20 ratio is calculated as the income earned by the top 20 % of households divided by the income earned by the poorest 20 % of households. Decile ratios (90/10, 80/20 and 50/50) are known to be simple and effective measures of income inequality. We used the 80/20 ratio according to previous studies [17].

### Statistical analysis

All statistical analyses except scatter plots were performed using SPSS version 15.0 (SPSS, Inc., Chicago, IL, USA). Scatter plots of all-cause mortality versus income inequality measures (GC, RHI, and 80/20 ratio) were graphed using Stata version 10 (Stata Corp, College Station, TX, USA). Analysis of covariance (ANCOVA) was used to compare mean all-cause mortality rates according to the quartiles of income inequality measures. Model 1 was not adjusted for any variable. Model 2 was adjusted for community population, percent of over 65 years old in community population (%), ratio of social welfare expenditure to the general budget, number of physicians per 1,000 individuals, smoking rate, and drinking rate. Model 3 was adjusted for Model 2 variables plus median household income. Statistical significance was set at *p* < 0.05.

## Results

### General characteristics of urban communities

Table 1 shows general characteristics of the 157 urban communities. The median value of GC, RHI, and 80/20 ratio were 0.318, 0.233, and 6.570, respectively. The median value of all-cause mortality per 100,000 was 416.3 and that of the community population was 280,277.0.

**Table 1:** General characteristics of urban communities

Variable	25th percentile	50th percentile	75th percentile	Standard deviation
GC	0.298	0.318	0.353	0.041
RHI	0.218	0.233	0.260	0.033
80/20 ratio	5.550	6.570	8.155	1.965
All-cause mortality rate (/100,000)	378.5	416.3	441.7	48.8
Community population	165,980.8	280,277.0	363,011.0	143,540.9
Percent of over 65 years in community population (%)	8.9	10.8	13.8	4.1
Ratio of social welfare expenditure (%)	23.9	30.9	44.6	12.2
Number of physicians (/1,000)	1.6	2.0	2.8	2.5
Smoking rate (%)	22.0	24.0	25.0	2.7
Drinking rate (%)	56.0	59.0	61.0	4.9

*GC*; Gini coefficient, *RHI*; Robin Hood index, *80/20 ratio*; 80th:20th percentile share ratio

### Correlation of income inequality measures with all-cause mortality

**Fig. 1:**
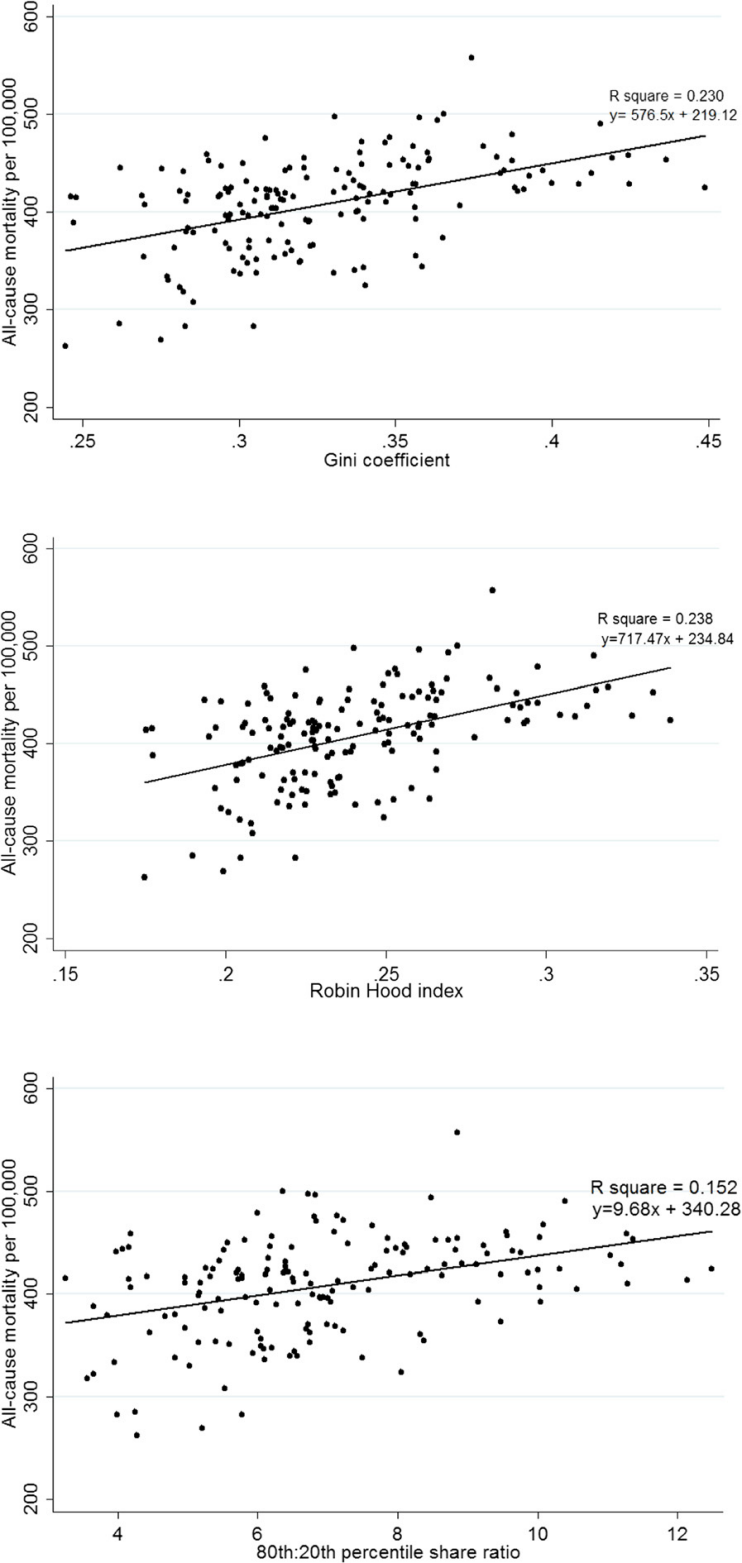
Scatter plot between all-cause mortality and income inequality measures

When we graphed income inequality measures versus all-cause mortality as scatter plots, the R square values of the regression lines for GC, RHI, and 80/20 ratios relative to mortality were 0.230, 0.238, and 0.152, respectively (Fig. 1).

### All-cause mortality rates according to quartiles of income inequality measures

Table 2 lists all-cause mortality rates according to quartiles of income inequality measures. After adjusting for covariates such as community population, percent of over 65 years old in community population, ratio of social welfare expenditure to the general budget, number of physicians per 1,000 individuals, and smoking and drinking rates (Model 2), mean all-cause mortality increased significantly with increasing income inequality (GC: *P* for trend < 0.001, RHI: *P* for trend < 0.001, 80/20 ratio: *P* for trend < 0.001). After additionally adjusting for median household income (Model 3), mean all-cause mortality increased significantly with increasing GC (*P* for trend = 0.014) and RHI (*P* for trend = 0.031), and increased marginally with 80/20 ratio (*P* for trend = 0.067).

**Table 2:** All-cause mortality rates according to quartiles of income inequality measures

Income inequality measures		Model 1^a^	Model 2^b^	Model 3^c^
GC	Q1 (0.245–0.298)	382.9 (369.0–396.8)	382.5 (369.0–396.1)	398.4 (386.5–410.4)
	Q2 (0.299–0.317)	392.5 (378.7–406.4)	394.8 (383.1–406.5)	396.4 (386.6–406.2)
	Q3 (0.318–0.353)	412.6 (398.9–426.3)	413.4 (402.6–424.2)	407.6 (398.4–416.8)
	Q4 (0.354–0.449)	441.1 (427.3–455.0)	440.1 (424.2–456.1)	428.1 (414.5–441.8)
	*P* for trend	<0.001	<0.001	0.014
RHI	Q1 (0.175–0.218)	379.6 (366.0–393.2)	381.3 (367.8–394.9)	400.7 (388.3–413.1)
	Q2 (0.219–0.233)	395.6 (382.0–409.3)	394.5 (383.2–405.9)	395.9 (386.3–405.5)
	Q3 (0.234–0.260)	410.1 (396.4–423.7)	412.6 (401.8–423.3)	406.3 (397.1–415.5)
	Q4 (0.261–0.339)	443.1 (429.6–456.6)	441.7 (426.3–457.2)	427.1 (413.6–440.6)
	*P* for trend	<0.001	<0.001	0.031
80/20 ratio	Q1 (3.25–5.53)	379.2(364.–393.)	387.9 (375.9–399.8)	399.6 (389.1–410.0)
	Q2 (5.57–6.56)	400.9(386.–415.)	405.8 (394.4–417.2)	405.7 (396.1–415.2)
	Q3 (6.57–8.13)	416.1(402.–430.)	411.0 (400.2–421.8)	410.7 (401.6–419.7)
	Q4 (8.18–12.49)	432.9(418.–447.)	426.2 (413.0–439.4)	414.4 (403.0–425.9)
	*P* for trend	<0.001	<0.001	0.067

*GC*; Gini coefficient, *RHI*; Robin Hood index, *80/20 ratio*; 80th:20th percentile share ratio

^a^Non-adjusted

^b^Adjusted by community population, Percent of over 65 years in community population (%), ratio of social welfare expenditure to the general budget, number of physicians per 1,000 population, smoking and drinking rate median household income

^c^Adjusted by Model 2 variables plus median household income

## Discussion

This study examined whether income inequality measures are associated with all-cause mortality across urban communities in Korea. The results suggest that income inequality is positively associated with all-cause mortality after adjustment for covariates, including median household income.

Previous studies have found a significant association between income inequality and health indicators [3–7, 9, 18], while others have reported no significant relationship between the two [8, 12, 19, 20], and still others find that the association varies among subgroups [10, 21]. In our results, the significant association between income inequality measures and all-cause mortality remained after adjustment for median household income. However, it is difficult to directly compare these studies because they differ greatly in population characteristics, geographic units of analysis, methods of analysis, and measures of income and inequality [7].

Several plausible mechanisms may link income inequality and health. First, income inequality limits public spending on infrastructure and important services that promote health such as education, public welfare, health care, highways, the environment, and housing [22]. Second, wide income disparities intensify social hierarchies, increasing class conflict and reducing social cohesion and social capital such as civic trust and associated activities that promote health [23]. Third, income inequality is related to psychosocial processes that are damaging to health, such as perception of a low position on the socioeconomic hierarchy [24]. These mechanisms are not mutually exclusive but rather are likely to reinforce each other, and may operate to a greater or lesser extent at different geographic levels of income inequality [18].

A variety of income inequality measures have been used in previous studies. While GC is the most popular measure, the Atkinson index, coefficient of variation, decile ratios, RHI, proportional total income earned, Sen poverty measure, generalized entropy index, and Kakwani progressive index have also been used [25]. Studies on whether the choice of income inequality measure affect conclusions on the health effects of income inequality have yielded conflicting results [4, 26]. In a cross-sectional ecological study in the United States [3], RHI was strongly associated with cause-specific mortality, while GC was not. In our data, the R square value of the regression line for RHI versus all-cause mortality was greater than that for other income inequality measures (0.238 versus 0.230 for GC and 0.152 for 80/20 ratio). However, income inequality measures are not directly comparable because they each provide a qualitatively different perspective [25]. For example, GC is more closely correlated with the proportion of income received by households in the poorest decile and RHI is correlated with income received by most of the population [3].

Because of its rapid economic development, inequality issues are drawing increasing concern in Korea. Inequality including disparities in health status has become more serious after the foreign currency crisis in 1997, which intensified polarization of income and wealth [13]. As most research has focused on health disparities according to SES [27], few have examined them across regions. To our knowledge, only one study [28] investigated regional health disparities, and found that the association between GC and self-rated health was significant across 16 regions (the largest administrative district level) in Korea. Our examination on the community level, the smallest municipal unit capable of autonomous policy implementation, found a similar association.

This study had some limitations. First, we used only contextual variables. The contextual approach in the study of health behaviors and outcome might have statistical artifacts [29]. Further study should attempt to incorporate multi-level analysis of income inequality and mortality. Second, whether we adjusted for appropriate demographic variables that may also influence health status is unclear, as no consensus on this question has yet been reached. Other inequality studies have adjusted for such variables as social capital, unemployment, perceived control and the proportion of the population without a high school education. While we adjusted for those variables for which data was available, other factors may still have confounding effects.

In conclusion, our data demonstrate that income inequality measures are significantly associated with all-cause mortality across urban communities in Korea.

## Abbreviations

KSIS: Korean Statistical Information Service
KCHS: Korean Community Health Survey
KCDC: Korean Centers for Disease Control and Prevention
GC: Gini coefficient
RHI: Robin Hood index
80/20 ratio: Income share ratio between the 80th and 20th percentiles of the distribution
ANCOVA: Analysis of covariance

## References

[1] Wilkinson RG (1992). Income distribution and life expectancy. BMJ.

[2] Kennedy BP, Kawachi I, Glass R, Prothrow-Stith D (1998). Income distribution, socioeconomic status, and self rated health in the United States: multilevel analysis. BMJ.

[3] Kennedy BP, Kawachi I, Prothrow-Stith D (1996). Income distribution and mortality: cross sectional ecological study of the Robin Hood index in the United States. BMJ.

[4] Lynch JW, Kaplan GA, Pamuk ER, Cohen RD, Heck KE, Balfour JL (1998). Income inequality and mortality in metropolitan areas of the United States. Am J Public Health.

[5] Elgar FJ (2010). Income inequality, trust, and population health in 33 countries. Am J Public Health.

[6] Collison D, Dey C, Hannah G, Stevenson L (2007). Income inequality and child mortality in wealthy nations. J Public Health.

[7] Massing MW, Rosamond WD, Wing SB, Suchindran CM, Kaplan BH, Tyroler HA (2004). Income, income inequality, and cardiovascular disease mortality: relations among county populations of the United States, 1985 to 1994. South Med J.

[8] Auger N, Zang G, Daniel M (2009). Community-level income inequality and mortality in Québec, Canada. Public Health.

[9] Stanistreet D, Scott-Samuel A, Bellis MA (1999). Income inequality and mortality in England. J Public Health Med.

[10] Regidor E, Calle ME, Navarro P, Domínguez V (2003). Trends in the association between average income, poverty and income inequality and life expectancy in Spain. Soc Sci Med.

[11] De Vogli R, Mistry R, Gnesotto R, Cornia GA (2005). Has the relation between income inequality and life expectancy disappeared? Evidence from Italy and top industrialised countries. J Epidemiol Community Health.

[12] Shibuya K, Hashimoto H, Yano E (2002). Individual income, income distribution, and self rated health in Japan: cross sectional analysis of nationally representative sample. BMJ.

[13] Khang YH, Lynch JW, Kaplan GA (2005). Impact of economic crisis on cause-specific mortality in South Korea. Int J Epidemiol.

[14] Korean Statistical Information Service. Statistics Korea, Daejeon. 2014. http://kosis.kr/statisticsList/statisticsList_01List.jsp?vwcd=MT_ZTITLE&parentId=D#SubCont. Accessed 11 Apr 2014.

[15] Ryu SY, Park J, Choi SW, Han MA (2014). Associations Between Socio-demographic Characteristics and Healthy Lifestyles in Korean Adults: The Result of the 2010 Community Health Survey. J Prev Med Public Health.

[16] Atkinson AB, Micklewright J. Economic Transformation in Eastern Europe and the Distribution of Income. Cambridge, England: Cambridge University Press;1992

[17] Wilkinson R, Pickett K (2009). The Spirit Level: Why Greater Equality Makes Societies Stronger.

[18] Lochner K, Pamuk E, Makuc D, Kennedy BP, Kawachi I (2001). State-level income inequality and individual mortality risk: a prospective, multilevel study. Am J Public Health.

[19] Fiscella K, Franks P (1997). Poverty or income inequality as predictor of mortality: longitudinal cohort study. BMJ.

[20] Osler M, Prescott E, Grønbaek M, Christensen U, Due P, Engholm G (2002). Income inequality, individual income, and mortality in Danish adults: analysis of pooled data from two cohort studies. BMJ.

[21] Ross NA, Dorling D, Dunn JR, Henriksson G, Glover J, Lynch J (2005). Metropolitan income inequality and working-age mortality: a cross-sectional analysis using comparable data from five countries. J Urban Health.

[22] Dunn J, Burgess B, Ross N (2005). Income distribution, public services expenditures, and all cause mortality in US states. J Epidemiol Community Health.

[23] Kawachi I, Kennedy BP (1997). Health and social cohesion: why care about income inequality?. BMJ.

[24] Kawachi I, Kennedy BP (1999). Income inequality and health: pathways and mechanisms. Health Serv Res.

[25] Maio FGD (2007). Income inequality measures. J Epidemiol Community Health.

[26] Kawachi I, Kennedy BP (1997). The relationship of income inequality to mortality: Does the choice of indicator matter?. Soc Sci Med.

[27] Khang YH (2007). Historical Advances in Health Inequality Research. J Prev Med Public Health.

[28] Hong E, Ahn BC (2011). Income-related health inequalities across regions in Korea. Int J Equity Health.

[29] Jen MH, Jones K, Johnston R (2009). Compositional and contextual approaches to the study of health behaviour and outcomes: Using multi-level modelling to evaluate Wilkinson’s income inequality hypothesis. Health Place.

